# The Biological Variation of N-Terminal Pro-Brain Natriuretic Peptide in Postmenopausal Women with Type 2 Diabetes: A Case Control Study

**DOI:** 10.1371/journal.pone.0047191

**Published:** 2012-11-09

**Authors:** Susana González, Eric S. Kilpatrick, Stephen L. Atkin

**Affiliations:** 1 Diabetes Department, Bradford Teaching Hospitals NHS Foundation Trust, Bradford, West Yorkshire, United Kingdom; 2 Department of Clinical Biochemistry, Hull Royal Infirmary, Hull, East Riding of Yorkshire, United Kingdom; 3 Department of Medicine, University of Hull, Hull, East Riding of Yorkshire, United Kingdom; John Hunter Hospital, Australia

## Abstract

**Background:**

The incidence of heart failure in type 2 diabetes is high and it has poorer prognosis when compared with patients without diabetes. Access to echocardiography is limited and alternative methods to identify early heart failure such as the measurement of natriuretic peptides levels have been proposed. However, their wide biological variation could limit their clinical utility. Our aim was to determine if the intrinsic biological variation of one of these peptides, N-terminal proBNP, is as wide in type 2 diabetes as it is in health and to calculate the critical difference values that could be utilised in clinical practice to ensure changes observed between two samples are due to intervention rather than to its biological variability.

**Methodology/Principal Findings:**

12 postmenopausal women with diet controlled type 2 diabetes and without heart failure were compared with 11 control postmenopausal women without diabetes. N-terminal proBNP levels were measured on 10 occasions. The biological variation was calculated according to Fraser's methods. The mean NT-proBNP level was similar in both groups (mean ± standard deviation; type 2 diabetes, 10.7 pmol/L± 8.5 versus 8.49±6.0 pmol/L, p = 0.42). The biological variation was also similarly wide. The critical difference in patients with type 2 diabetes was between −70% and ±236%.

**Conclusions:**

Type 2 diabetes does not appear to significantly influence the marked biological variation of N-terminal proBNP in postmenopausal women. The critical difference values reported in this study could be used to titrate therapy or monitor response to interventions although the change required in between samples is wide and this might limit its utility.

## Introduction

The overall incidence rate of heart failure (HF) in patients with type 2 diabetes is 2.5 times higher than in patients without diabetes although this incidence is variable depending on the subgroup studied [1]. Type 2 diabetes is an independent predictor of morbidity and mortality in patients with HF [2]. Therefore, early identification of HF would be advantageous so the appropriate therapeutic measures could be implemented.

Echocardiography is the gold standard investigation to confirm suspected HF on clinical grounds but universal access is limited due to manpower and cost restrictions, hence surrogate biomarkers such as brain natriuretic peptide (BNP) and N-terminal proBNP (NT-proBNP) have been proposed in the literature as an alternative.

NT-proBNP, one of these natriuretic peptides, is the biologically inactive fraction that results after a protease splits the precursor proBNP secreted by the ventricular myocytes in response to tension and stretch. The active fraction, BNP, acts as counter regulatory hormone to the renin angiotensin system promoting balanced vasodilatation. Both fractions circulate in plasma and can be quantified by immunoassay. The advantage of measuring NT-proBNP is that is more stable than BNP likely related to its physiological release and clearance. It has a half-life between 1 and 2 hours and greater reliance on glomerular filtration because it lacks a clearance receptor that metabolises it [3], [4].

Natriuretic peptides have been considered to aid the exclusion of HF in borderline cases due to their negative predictive value and to facilitate patient selection for echocardiography which might be cost effective particularly in primary care. They have a strong association with total mortality [5], [6], can predict further cardiac events after an acute episode [7] and their levels are proportional to the severity of HF, correlating with the New York Heart Association function class [8], [9], [10] hence they are also useful to evaluate HF prognosis. Their utility could be especially advantageous in postmenopausal women since they lose their cardiovascular protection when menopause is attained placing them at higher risk of developing cardiovascular disease and heart failure [11].

However, their value to monitor disease progression and/or response to treatment has been questioned due to their marked biological variation (BV) reported both in health [12] and in chronic HF [13], [14]. This BV refers to the natural fluctuation of NT-proBNP around a homeostatic setting point regulated by complex neurohormonal and haemodynamic stimuli that control NT-proBNP secretion and clearance. This fluctuation may occur either in a random fashion and/or in a more predictable pattern and comprises two components: the within person or intra-individual variation (the variation around a homeostatic setting point in an individual) and in between person or inter-individual variation (the variation around a homeostatic setting point among different subjects). The BV together with the preanalytical (specimen collection), analytical (imprecision and bias) and postanalytical (reporting of the results) variations are the main sources of variability that can influence any laboratory test, in this case NT-proBNP, and may limit its clinical utility if not adequately controlled. Awareness of this natural fluctuation is essential to appropriately obtain specimens that reflect the clinical scenario studied, for example, the timing of the sample following the initiation of HF therapy.

Additionally, a limited number of studies have reported, with conflicting results, that diabetes might alter the intrinsic variability of these natriuretic peptides [15]–[17].

The aim of this study was to establish whether NT-proBNP levels in postmenopausal women with type 2 diabetes remain within narrow biological limits or vary more widely over time when compared with a matched control group of postmenopausal women without diabetes. This information could be used to derive the critical difference values that are essential for an accurate interpretation of any change between serial measurements.

## Methods

### Objectives

The objectives of this study were to compare the biological variation of NT-proBNP in postmenopausal women with and without diet controlled type 2 diabetes and to calculate the critical difference values that permit the interpretation of NT-proBNP serial results.

### Participants

Twelve obese, Caucasian, postmenopausal women with diet controlled type 2 diabetes diagnosed according to WHO criteria [18] were weight matched with eleven obese Caucasian postmenopausal women with normal fasting blood glucose levels and not known comorbidities or on medication. The subjects were recruited over a 4 week period.

Women were considered postmenopausal if they had amenorrhoea for >1 year and Follicle Stimulating hormone (FSH) levels >20 IU/L. All the patients were advised by a registered dietician to follow the British Heart Foundation guidelines on Healthy Eating.

Exclusion criteria included secondary causes of hyperglycaemia, oestrogen therapy, hypertension, untreated hypothyroidism, history of drug, alcohol abuse or smoking, anaemia, atrial fibrillation and HF (excluded by clinical findings and echocardiography). All the patients had an estimated Glomerular Filtration Rate (eGFR) >90 mL/min/1.73 m2. These patients were recruited from a tertiary centre.

### Description of Procedures

Venous blood samples were obtained on 10 consecutive occasions at 4 days intervals following 12 hours overnight fast, between 8–9 am and after 10 minutes rest. Samples were separated by centrifugation at 2000 g for 15 minutes at 4°C and the serum obtained was stored in two aliquots at –20°C within one hour of collection. The serum samples were split before the assay. All the serum samples were thawed and thoroughly mixed before the analysis. The duplicate samples (i.e. two per visit) were randomized and then analysed in a single continuous batch using a single batch of reagents.

NT-proBNP serum levels were quantified with an electrochemiluminescence immunoassay used on the Roche Elecsys® Systems 1010/2010. The analytical sensitivity was 0.6pmol/L with a measuring range between 0.6 and 4130 pmol/L .The within run precision with this method is 1.8%. The assay is unaffected by bilirubin, haemolysis or lipaemia with no stated cross-reactions with Atrial natriuretic peptide (ANP), Nterminal-proANP (NT-proANP), BNP, C-type natriuretic peptide (CNP), aldosterone, angiotensin (I, II, III) or renin.

### Ethics

This study followed the declaration of Helsinki guidelines and it was approved by the Hull and East Riding Local Research Ethics Committee. All patients provided written, informed consent.

### Statistical methods

Biovariability data was analysed over two weeks by calculating analytical, within subject, and between subject variances (SDA^2^, SDI^2^, SDG^2^, respectively) according to the methods of Fraser and Co- workers [19], [20]. By this technique, analytical variance (SDA^2^) was calculated from the difference between duplicate results for each specimen (SDA^2^  =  Σd2/2N, where d is the difference between duplicates, and N is the number of paired results). The variance of the first set of duplicate results for each subject on the ten assessment days was used to calculate the average biological intra-individual variance (SDI^2^) by subtraction of SDA^2^ from the observed dispersion (equal to SDI^2^ + SDA^2^). Subtracting SDI^2^ + SDA^2^ from the overall variance of the set of first results determined the inter-individual variance (SDG^2^). The intra-individual (SDI) and inter-individual (SDG) variations were estimated as square roots of the respective variance component estimates. The reference change value (RCV) or critical difference between two consecutive samples in an individual subject (i.e., the smallest percentage change unlikely to be due to biological variability) was calculated using the formula RCV  = 2.77*(CVA^2^ + CVI^2^)½ where CVI is the within subject biological coefficient of variation and CVA is the analytical coefficient of variation.

It remains unclear the precise number of subjects required to undertake biological variation studies [19], [20]. Therefore, the sample size and number of repeated measures used were based on previous studies [21]–[28] investigating biological variation in measured analytes suggesting that a minimum of eight subjects are needed. To account for the potential drops out due to the intensity of the study, twelve subjects in the diabetic group and eleven in the non-diabetic group were recruited. The mean values of NT-proBNP were found to be non Gaussian (by Kolmogorov test) in both subgroups. This lack of central tendency was reduced following logarithmic transformation but not completely eliminated and therefore, it was analysed with the Mann Whitney U test using SPSS version 15.

## Results

**Figure 1:**
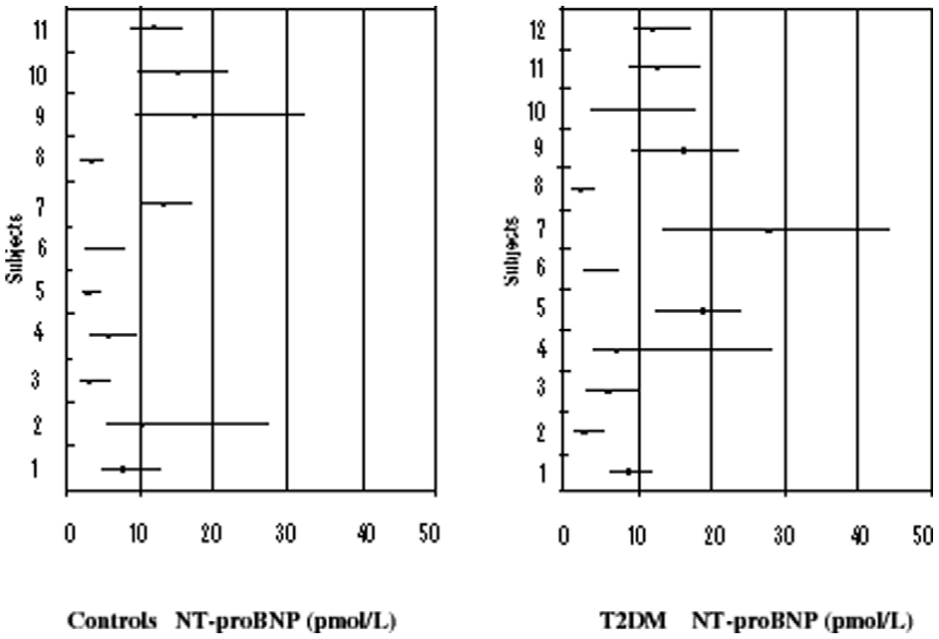
Means and range of values for NT-proBNP (unadjusted for analytical variation) in controls and subjects with type 2 diabetes (T2DM).

All the patients completed the study. Table 1. summarises the clinical and biochemical characteristics of the subjects. Figure 1. shows the means and range of values for NT-proBNP in subjects with and without diabetes.

**Table 1:** Clinical and biochemical features of study subjects.

Parameter	Type 2 Diabetes (n = 12)	Controls (n = 11)	P value
Age (years)	61.7±7.0	56.2±6.1	0.06
Weight (Kg)	77.5±8.1	79.9±13.4	0.60
BMI (Kg/m^2^)	31.1±3.3	32.4±5.3	0.49
Fasting glucose (mmol/L)	7.6±2.3	5.0±0.5	<0.001
NT-proBNP (pmol/L)	10.7±8.5	8.49±6.0	0.42

Data expressed as mean± Standard deviation. BMI: body mass index. All parameters but NT-proBNP levels were compared between the two groups with the Student's t-test because they were normally distributed. NT-proBNP levels were compared with the Mann Whitney U test.

The mean NT-proBNP level was similar in both groups (mean ± standard deviation; type 2 diabetes, 10.7 pmol/L±8.5 versus 8.49±6.0 pmol/L, p = 0.42). In the control group, the analytical variance contributed 7.9% of the total variance. After accounting for the analytical variation, the intraindividual and interindividual variances contributed 21.4% and 29% respectively. The critical difference for sequential NT-proBNP values was between −67% and +204%.

In the group with type 2 diabetes, analytical variance contributed 7.07% of the total variance and after adjusting for the analytical variation, the intraindividual variance was 18.3% and the interindividual variance contributed 26.6%. The critical difference was between −70% and +236%.

## Discussion

This is the first study to show that the absolute NT-proBNP value and its biological variation are as wide in obese postmenopausal women with diet controlled type 2 diabetes as in obese postmenopausal women without diabetes, suggesting that type 2 diabetes perse, at least in the early stages, does not significantly alter NT-proBNP levels in this group of patients. This wide natural variation needs to be accounted for the interpretation of serial results and the critical difference values provided could be used to assess the significance of any change between two consecutive measurements of NT-proBNP. These values suggests that small changes between two single measurements maybe more of a consequence of the biological variation rather than the reflection of therapeutic interventions or disease progression because they must rise by more than 236% or fall by greater than 70% before any critical difference can be assured.

Previous studies have evaluated the biological variation of NT-proBNP in health and in stable chronic HF. Although NT-proBNP secretion does not appear to follow a circadian rhythm [29], it has a substantial intraindividual variation in health with suggested reference change values ranging between 26% [12] and 90% [30]. In chronic heart failure, this high intraindividual variation remains and changes in values from week to week of up to 98% are required to indicate a change in clinical status[13], [14].

NT- proBNP level is influenced not only by its biological variation but also by several factors that may modify its value. Raised natriuretic peptide levels are associated with heart failure, atrial fibrillation [31], acute coronary syndrome, pulmonary embolism, hypertension, renal dysfunction [32] and anaemia [33], [34]. In addition, advanced age and female sex also appear to increase levels [35] while obesity reduces them [36] although the exact physiological mechanisms responsible for this remain unclear. The cohorts included in the biological variation studies are mixed, with a variable male/female ratio and age range and may partially explain the different percentage of change required.

This heterogeneity in subjects selection appears to be also present in a recent systematic review evaluating studies that utilised natriuretic peptides as tools to monitor response to therapy in patients with various degrees of heart failure. They concluded that there was limited data available to support the use of NT-proBNP or BNP in this context [37]. To avoid all these confounding factors, our postmenopausal subjects were age, gender and BMI matched and indeed, the biological variation was wide with large reference change values both in health and in type 2 diabetes.

One of the implications of the findings reported here is that current consensus guidelines [38], [39] that provide recommendations to use natriuretic peptides in both hospital and community settings in the general population could be extrapolated to postmenopausal women with type 2 diabetes since the biological variation of NT-proBNP is similar to that in health.

### Limitations

The key limitation of this study is that the results obtained are applicable to a specific cohort of patients (obese, postmenopausal women with type 2 diabetes treated with diet alone) and these findings might differ in other subjects with different characteristics, for example, slim males with type 2 diabetes treated with insulin.

In conclusion, type 2 diabetes does not appear to significantly influence the marked biological variation of NT-proBNP in postmenopausal women. The critical difference values reported in this study could be used to titrate therapy or monitor response to interventions although the change required in between samples is wide and this might limit its utility. Further studies are required to clarify the need to use standard or individualised targets based on age, sex and body weight and to determine the frequency of testing.

## References

[1] Nichols GA, Gullion CM, Koro CE, Ephross SA, Brown JB (2004) The incidence of congestive heart failure in type 2 diabetes: an update. Diabetes Care 27: 1879–1884. 15277411 10.2337/diacare.27.8.1879

[2] Shindler DM, Kostis JB, Yusuf S, Quinones MA, Pitt B, et al (1996) Diabetes mellitus, a predictor of morbidity and mortality in the Studies of Left Ventricular Dysfunction (SOLVD) Trials and Registry. Am J Cardiol 77: 1017–1020. 8644628 10.1016/s0002-9149(97)89163-1

[3] Hall C (2004) Essential biochemistry and physiology of (NT-pro)BNP. Eur J Heart Fail 6: 257–260. 14987573 10.1016/j.ejheart.2003.12.015

[4] Wu AH, Smith A (2004) Biological variation of the natriuretic peptides and their role in monitoring patients with heart failure. Eur J Heart Fail 6: 355–358. 14987588 10.1016/j.ejheart.2003.12.011

[5] Anand IS, Fisher LD, Chiang YT, Latini R, Masson S, et al (2003) Changes in brain natriuretic peptide and norepinephrine over time and mortality and morbidity in the Valsartan Heart Failure Trial (Val-HeFT). Circulation 107: 1278–1283. 12628948 10.1161/01.cir.0000054164.99881.00

[6] Hartmann F, Packer M, Coats AJ, Fowler MB, Krum H, et al (2004) NT-proBNP in severe chronic heart failure: rationale, design and preliminary results of the COPERNICUS NT-proBNP substudy. Eur J Heart Fail 6: 343–350. 14987586 10.1016/j.ejheart.2004.01.009

[7] Harrison A, Morrison LK, Krishnaswamy P, Kazanegra R, Clopton P, et al (2002) B-type natriuretic peptide predicts future cardiac events in patients presenting to the emergency department with dyspnea. Ann Emerg Med 39: 131–138. 11823766 10.1067/mem.2002.121483

[8] Jourdain P, Funck F, Bellorini M, Guillard N, Loiret J, et al (2003) Bedside B-type natriuretic peptide and functional capacity in chronic heart failure. Eur J Heart Fail 5: 155–160. 12644005 10.1016/s1388-9842(02)00247-7

[9] Koglin J, Pehlivanli S, Schwaiblmair M, Vogeser M, Cremer P, et al (2001) Role of brain natriuretic peptide in risk stratification of patients with congestive heart failure. J Am Coll Cardiol 38: 1934–1941. 11738297 10.1016/s0735-1097(01)01672-2

[10] Song BG, Jeon ES, Kim YH, Kang MK, Doh JH, et al (2005) Correlation between levels of N-terminal pro-B-type natriuretic peptide and degrees of heart failure. Korean J Intern Med 20: 26–32. 15906950 10.3904/kjim.2005.20.1.26 PMC3891409

[11] Barrett-Connor E (2003) Clinical review 162: cardiovascular endocrinology 3: an epidemiologist looks at hormones and heart disease in women. J Clin Endocrinol Metab 88: 4031–4042. 12970259 10.1210/jc.2003-030876

[12] Melzi d'Eril G, Tagnochetti T, Nauti A, Klersy C, Papalia A, et al (2003) Biological variation of N-terminal pro-brain natriuretic peptide in healthy individuals. Clin Chem 49: 1554–1555. 12928248 10.1373/49.9.1554

[13] Bruins S, Fokkema MR, Romer JW, Dejongste MJ, van der Dijs FP, et al (2004) High intraindividual variation of B-type natriuretic peptide (BNP) and amino-terminal proBNP in patients with stable chronic heart failure. Clin Chem 50: 2052–2058. 15345664 10.1373/clinchem.2004.038752

[14] O'Hanlon R, O'Shea P, Ledwidge M, O'Loughlin C, Lange S, et al (2007) The biologic variability of B-type natriuretic peptide and N-terminal pro-B-type natriuretic peptide in stable heart failure patients. J Card Fail 13: 50–55. 17339003 10.1016/j.cardfail.2006.09.003

[15] Yano Y, Katsuki A, Gabazza EC, Ito K, Fujii M, et al (1999) Plasma brain natriuretic peptide levels in normotensive noninsulin-dependent diabetic patients with microalbuminuria. J Clin Endocrinol Metab 84: 2353–2356. 10404802 10.1210/jcem.84.7.5819

[16] Magnusson M, Melander O, Israelsson B, Grubb A, Groop L, et al (2004) Elevated plasma levels of Nt-proBNP in patients with type 2 diabetes without overt cardiovascular disease. Diabetes Care 27: 1929–1935. 15277419 10.2337/diacare.27.8.1929

[17] Wu AH, Omland T, Duc P, McCord J, Nowak RM, et al (2004) The effect of diabetes on B-type natriuretic peptide concentrations in patients with acute dyspnea: an analysis from the Breathing Not Properly Multinational Study. Diabetes Care 27: 2398–2404. 15451907 10.2337/diacare.27.10.2398

[18] World Health Organization (1999) Definition, Diagnosis and Classification of Diabetes Mellitus and its complications. In: surveillance Doncd, editor: World Health Organization. Geneva.

[19] Fraser CG (2001) Biological variation: from principles to practice. Washington DC: AACC Press. 151 p.

[20] Fraser CG, Harris EK (1989) Generation and application of data on biological variation in clinical chemistry. Crit Rev Clin Lab Sci 27: 409–437. 2679660 10.3109/10408368909106595

[21] Keevil BG, Kilpatrick ES, Nichols SP, Maylor PW (1998) Biological variation of cystatin C: implications for the assessment of glomerular filtration rate. Clin Chem 44: 1535–1539. 9665434

[22] Kilpatrick ES, Maylor PW, Keevil BG (1998) Biological variation of glycated hemoglobin. Implications for diabetes screening and monitoring. Diabetes Care 21: 261–264. 9539993 10.2337/diacare.21.2.261

[23] Jayagopal V, Kilpatrick ES, Holding S, Jennings PE, Atkin SL (2002) The biological variation of insulin resistance in polycystic ovarian syndrome. J Clin Endocrinol Metab 87: 1560–1562. 11932282 10.1210/jcem.87.4.8404

[24] Jayagopal V, Kilpatrick ES, Jennings PE, Hepburn DA, Atkin SL (2002) Biological variation of homeostasis model assessment-derived insulin resistance in type 2 diabetes. Diabetes Care 25: 2022–2025. 12401750 10.2337/diacare.25.11.2022

[25] Jayagopal V, Kilpatrick ES, Jennings PE, Hepburn DA, Atkin SL (2003) The biological variation of testosterone and sex hormone-binding globulin (SHBG) in polycystic ovarian syndrome: implications for SHBG as a surrogate marker of insulin resistance. J Clin Endocrinol Metab 88: 1528–1533. 12679434 10.1210/jc.2002-020557

[26] Cho LW, Jayagopal V, Kilpatrick ES, Atkin SL (2005) The biological variation of C-reactive protein in polycystic ovarian syndrome. Clin Chem 51: 1905–1907. 16189386 10.1373/clinchem.2005.052753

[27] Browne RW, Bloom MS, Schisterman EF, Hovey K, Trevisan M, et al (2008) Analytical and biological variation of biomarkers of oxidative stress during the menstrual cycle. Biomarkers 13: 160–183. 18270869 10.1080/13547500701775563 PMC2504010

[28] Cho LW, Jayagopal V, Kilpatrick ES, Atkin SL (2009) The mean and the biological variation of insulin resistance does not differ between polycystic ovary syndrome and type 2 diabetes. Ann Clin Biochem 46: 218–221. 19389885 10.1258/acb.2008.008146

[29] Sokoll LJ, Baum H, Collinson PO, Gurr E, Haass M, et al (2004) Multicenter analytical performance evaluation of the Elecsys proBNP assay. Clin Chem Lab Med 42: 965–972. 15387451 10.1515/CCLM.2004.157

[30] Wu AH, Smith A, Wieczorek S, Mather JF, Duncan B, et al (2003) Biological variation for N-terminal pro- and B-type natriuretic peptides and implications for therapeutic monitoring of patients with congestive heart failure. Am J Cardiol 92: 628–631. 12943894 10.1016/s0002-9149(03)00741-0

[31] Knudsen CW, Omland T, Clopton P, Westheim A, Wu AH, et al (2005) Impact of atrial fibrillation on the diagnostic performance of B-type natriuretic peptide concentration in dyspneic patients: an analysis from the breathing not properly multinational study. J Am Coll Cardiol 46: 838–844. 16139134 10.1016/j.jacc.2005.05.057

[32] Boomsma F, van den Meiracker AH (2001) Plasma A- and B-type natriuretic peptides: physiology, methodology and clinical use. Cardiovasc Res 51: 442–449. 11476734 10.1016/s0008-6363(01)00195-x

[33] Willis MS, Lee ES, Grenache DG (2005) Effect of anemia on plasma concentrations of NT-proBNP. Clin Chim Acta 358: 175–181. 15878465 10.1016/j.cccn.2005.03.009

[34] Wold Knudsen C, Vik-Mo H, Omland T (2005) Blood haemoglobin is an independent predictor of B-type natriuretic peptide (BNP). Clin Sci (Lond) 109: 69–74. 15755257 10.1042/CS20040349

[35] Raymond I, Groenning BA, Hildebrandt PR, Nilsson JC, Baumann M, et al (2003) The influence of age, sex and other variables on the plasma level of N-terminal pro brain natriuretic peptide in a large sample of the general population. Heart 89: 745–751. 12807847 10.1136/heart.89.7.745 PMC1767734

[36] Das SR, Drazner MH, Dries DL, Vega GL, Stanek HG, et al (2005) Impact of body mass and body composition on circulating levels of natriuretic peptides: results from the Dallas Heart Study. Circulation 112: 2163–2168. 16203929 10.1161/CIRCULATIONAHA.105.555573

[37] Balion CM, McKelvie RS, Reichert S, Santaguida P, Booker L, et al (2008) Monitoring the response to pharmacologic therapy in patients with stable chronic heart failure: is BNP or NT-proBNP a useful assessment tool? Clin Biochem 41: 266–276. 17991434 10.1016/j.clinbiochem.2007.10.006

[38] Silver MA, Maisel A, Yancy CW, McCullough PA, Burnett JC Jr, et al (2004) BNP Consensus Panel 2004: A clinical approach for the diagnostic, prognostic, screening, treatment monitoring, and therapeutic roles of natriuretic peptides in cardiovascular diseases. Congest Heart Fail 10: 1–30. 10.1111/j.1527-5299.2004.03271.x 15604859

[39] Troughton RW, Richards AM (2008) Outpatient monitoring and treatment of chronic heart failure guided by amino-terminal pro-B-type natriuretic peptide measurement. Am J Cardiol 101: 72–75. 18243863 10.1016/j.amjcard.2007.11.027

